# Early IFNγ-Mediated and Late Perforin-Mediated Suppression of Pathogenic CD4 T Cell Responses Are Both Required for Inhibition of Demyelinating Disease by CNS-Specific Autoregulatory CD8 T Cells

**DOI:** 10.3389/fimmu.2018.02336

**Published:** 2018-10-09

**Authors:** Alexander W. Boyden, Ashley A. Brate, Nitin J. Karandikar

**Affiliations:** ^1^Department of Pathology, University of Iowa, Iowa City, IA, United States; ^2^Iowa City Veterans Affairs Medical Center, Iowa City, IA, United States; ^3^Interdisciplinary Graduate Program in Immunology, University of Iowa Health Care, Iowa City, IA, United States

**Keywords:** CD8 T cells, multiple sclerosis, EAE, autoimmunity, regulation, CD4 T cells, interferon-gamma, perforin

## Abstract

Pathogenesis of immune-mediated demyelinating diseases like multiple sclerosis (MS) is thought to be governed by a complex cellular interplay between immunopathogenic and immunoregulatory responses. We have previously shown that central nervous system (CNS)-specific CD8 T cells have an unexpected protective role in the mouse model of MS, experimental autoimmune encephalomyelitis (EAE). In this study, we interrogated the suppressive potential of PLP178-191-specific CD8 T cells (PLP-CD8). Here, we show that PLP-CD8, when administered post-disease onset, rapidly ameliorated EAE progression, and suppressed PLP178-191-specific CD4 T cell responses as measured by delayed-type hypersensitivity (DTH). To accomplish DTH suppression, PLP-CD8 required differential production of perforin and IFNγ. Perforin was not required for the rapid suppressive action of these cells, but was critical for maintenance of optimal longer term DTH suppression. Conversely, IFNγ production by PLP-CD8 was necessary for swift DTH suppression, but was less significant for maintenance of longer term suppression. These data indicate that CNS-specific CD8 T cells employ an ordered regulatory mechanism program over a number of days *in vivo* during demyelinating disease and have mechanistic implications for this immunotherapeutic approach.

## Introduction

Multiple sclerosis (MS) is an immune-mediated demyelinating disease of the central nervous system (CNS), whereby infiltrating proinflammatory immune cells potentiate recruitment and continued activation of additional inflammatory cell types which target and destroy myelin (1). Despite the current first-line drug therapies available to patients as well as recent advancements in US clinical trials (2–4), MS remains a debilitating disease that worsens over time and for which there is no cure.

In order to dissect the dynamics of immunopathogenic and immunoregulatory responses during MS, researchers use the mouse model experimental autoimmune encephalomyelitis (EAE), which manifests as an ascending paralytic disease due to spinal cord demyelination (5). Given that CD4 T cells from EAE mice are sufficient to transfer disease to healthy animals (6, 7), the field has focused for many years on this encephalitogenic cell, its Th1 and Th17 pro-inflammatory states, and its role in driving demyelinating disease (8). The role of CD8 T cells however, which are oligoclonally expanded to large numbers in MS lesions (9, 10), is less well understood.

In multiple previous studies, we have now demonstrated the unexpected disease suppressive effect of CNS-specific CD8 T cells (CNS-CD8) in various models of EAE (7, 11–14). These “autoregulatory” CNS-CD8 are unlike the “typical” regulatory T cell populations in that they lack Foxp3 expression and do not depend on anti-inflammatory cytokine production (e.g., IL-4 or IL-10), but are dependent on classical MHC class Ia presentation and require elaboration of IFNγ and perforin (11, 14). Importantly, the clinical and therapeutic relevance of their role during demyelinating disease is underscored by the finding that MS patients undergoing an acute relapse have a defect in autoregulatory CD8 T cell function compared to disease quiescent patients or healthy controls (15, 16). Therefore, interrogating CNS-CD8 subsets' regulatory potential and *in vivo* suppression mechanisms during demyelinating disease is of high interest and importance.

We have demonstrated that CD8 T cells recognizing the encephalitogenic 178-191 peptide sequence of myelin proteolipid protein (PLP178-191) were superior suppressors of EAE disease compared to myelin oligodendrocyte glycoprotein (MOG) 35-55-specific CD8 T cells and were suppressive in different models of EAE (12–14). Given that PLP is the main structural component of the myelin sheath (50% of total protein) and murine and human forms share 100% amino acid sequence homology (17), we studied the *in vivo* therapeutic potential and mechanisms of PLP178-191-specific CD8 T cells (PLP-CD8) during EAE. Here, we show that PLP-CD8 swiftly ameliorated ongoing demyelinating disease and rapidly suppressed PLP-specific CD4 T cell responses by employing a temporally distinct cytokine effector program over a number of days *in vivo*.

## Materials and methods

### Mice

Wildtype female C57BL/J, perforin-/-, and IFNγ-/- mice were purchased from Jackson Laboratories (Bar Harbor, ME). All mice were kept in barrier rooms at the University of Iowa Animal Care Facility under 12 h light/dark cycle, fed *ad libitum*, and humanely cared for and studied as approved by the University of Iowa's Institutional Animal Care and Use Committee. All mice used in experiments were at least 8 weeks of age.

### Disease induction and evaluation

Disease was induced and evaluated as published previously (11, 12, 14). Briefly, mice were immunized s.c. on day 0 in the right and left flank with 100 μg PLP178-191 (NTWTTCQSIAFPSK, GenScript, Piscataway, NJ) emulsified 1:1 volume in complete Freund's adjuvant supplemented with 4 mg/ml *Mycobacterium tuberculosis* (CFA; Becton Dickinson, Franklin Lakes, NJ), followed by 250 ng pertussis toxin (PTx) i.p. on days 0 and 2. Clinical scores were assessed in a blinded manner by ascending paralysis scale: 0, no symptoms; 1, loss of tail tonicity; 2, partial hind limb weakness; 3, partial hind limb paralysis; 4, complete hind limb paralysis; 5, moribund or death.

### CD8 T cell adoptive transfer

Donor mice were immunized with either control OVA323-339 (ISQAVHAAHAEINEAGR, GenScript, Piscataway, NJ) or PLP178-191 in CFA. Splenocytes and inguinal lymphocytes were harvested 15–17 days post-immunization. As published previously (7, 11–13), single cell suspensions were stimulated *in vitro* with cognate antigen and rIL-2 for 72 h in complete RPMI (Corning, Tewksbury, MA). CD8 T cells were subsequently Ly-2 microbeadsorted (Miltenyi Biotech, Auburn, CA) to >90% purity, and 5 × 10^6^ cells were adoptively transferred i.v. into recipient mice at times indicated. For experiments containing a mixture of perforin- and IFNγ-deficient CD8 T cells, a total of 5 × 10^6^ cells were transferred (i.e., 2.5 × 10^6^ + 2.5 × 10^6^).

### Delayed-Type hypersensitivity (DTH)/Ear swelling assays

For DTH measurements, 15 μL of either vehicle (PBS) alone or 150 μg PLP178-191 in PBS were injected into ear pinnae of briefly anesthetized (isoflurane USP, Clipper Distributing, St. Joseph, MO) immune recipients with a 30G needle and 1cc syringe. DTH was elicited at various times depending on the experiment (e.g., at times on the same day as CD8 T cell adoptive transfer and others seven days post-transfer and still others 9 or 20 days post-immunization for EAE), as indicated in the figure legends. Ear swelling was measured in a blinded manner with an engineer's micrometer (Mitutoyo USA, Aurora, IL) on day of injection and at 24 or 48 h, as indicated. Delta ear swelling was calculated by ear thickness (mm) at 24/48 h minus thickness at 0 h. Where noted, data were normalized to control group mean when combining swelling measurements from separate experiments.

### Statistics

EAE scores from two groups were compared using a Welch's *t*-test. DTH measurements from multiple groups were compared using the ANOVA test. All statistics were calculated using GraphPad Prism software (La Jolla, CA). *P*-values < 0.05 were considered significant.

## Results

### PLP-Specific CD8 T cells downregulate CD4 T cell responses during demyelinating disease

**Figure 1:**
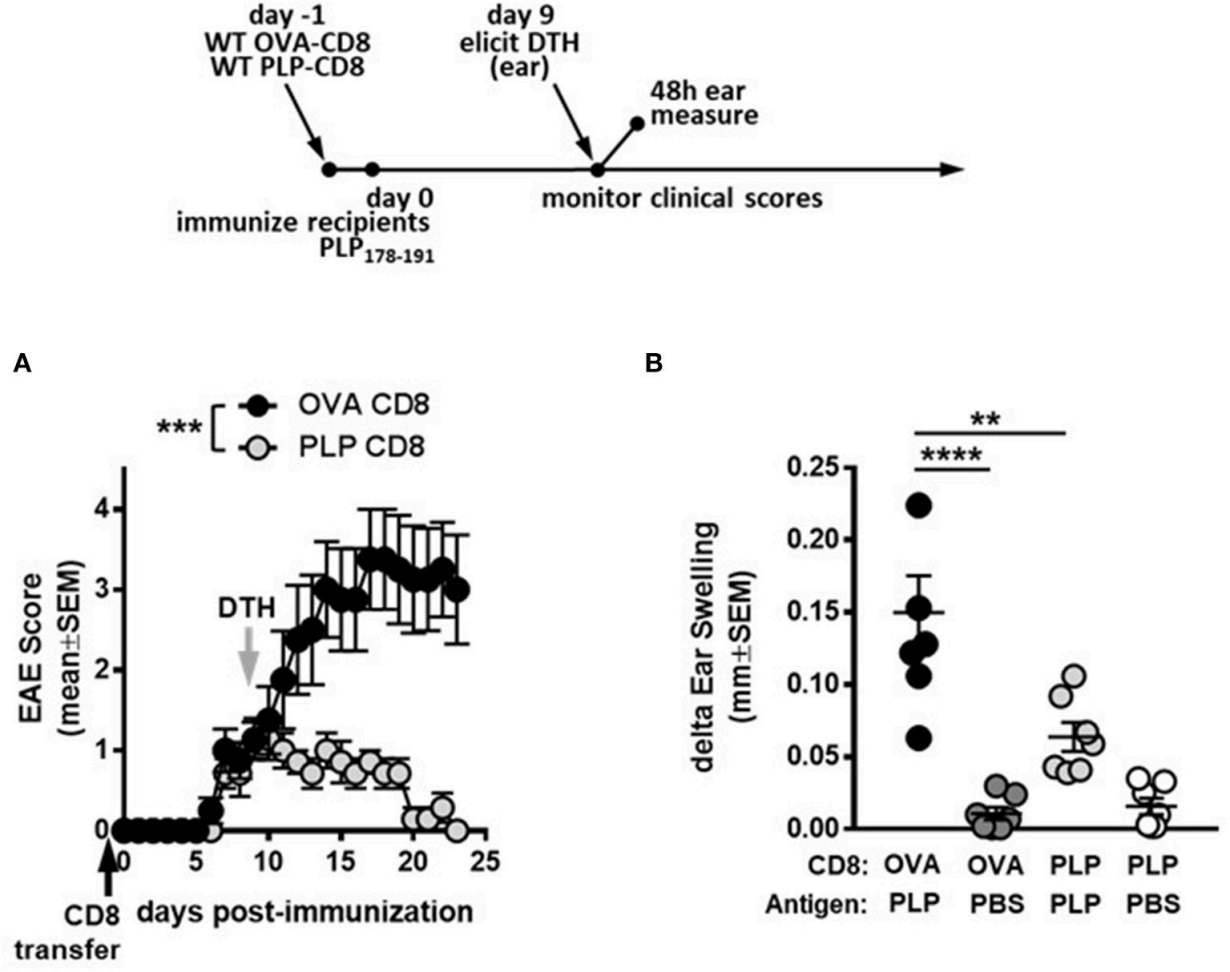
PLP-specific CD8 T cells suppress PLP-specific CD4 T cell responses during demyelinating disease. **(A)** On day−1 (black arrow), OVA-CD8 or PLP-CD8 were adoptively transferred to naïve recipients. The following day, mice were immunized with PLP178-191/CFA to induce EAE and disease scores were monitored. *n* = 7 per group. **(B)** On day 9 post-immunization (gray arrow in **A)**, ear pinnae were injected with either PLP178-191 peptide or vehicle control (PBS). Ear swelling was measured at 48 h post-ear challenge. *n* = 7 per group. **p < 0.01; ***p < 0.001; ****p < 0.0001.

First, to confirm the suppressive potential of PLP-CD8, donor CD8 T cells from OVA323-339- or PLP178-191-immunized mice were adoptively transferred into naïve C57BL/6J mice. The following day, recipient mice were immunized with PLP178-191/CFA to induce EAE and disease scores were monitored. Consistent with our previous observations, mice receiving PLP-CD8 were significantly protected from EAE disease compared to their OVA323-339-specific CD8 T cell (OVA-CD8)-transferred counterparts (Figure 1A). As previously demonstrated, disease scores in the OVA-CD8 control group were not different from disease control groups that received PBS or did not receive any treatment at all (data not shown).

We then tested the functional effects of PLP-CD8 treatment on *in vivo* readouts of CD4 function. Delayed type hypersensitivity (DTH) responses to CNS peptide antigens have been used as robust readouts of CD4 function (18–20). Importantly, DTH has also been used to assess suppressive fitness of regulatory CD8 T cell populations on CNS peptide MOG35-55 responses (21, 22). We therefore studied the ability of PLP-CD8 to downregulate CD4 T cell responses *in vivo* through a similar method.

To confirm CNS peptide-specific DTH responses in our system, mice were immunized with PBS/CFA, MOG35-55/CFA, or PLP178-191/CFA. For DTH response measurements, either PBS (vehicle control) or PLP178-191 peptide (in PBS) were injected into the pinnae of immunized mice. As expected, PBS injection resulted in minimal to no swelling. PLP178-191/CFA-immunized mice developed a robust DTH reaction to PLP178-191 peptide (Supplementary Figure 1) that was significantly greater than the PBS control ears, whereas neither PBS/CFA- nor MOG35-55/CFA-immunized mice developed DTH responses to PLP178-191, showing swelling that was comparable to PBS (Supplementary Figure 1). Given that DTH readouts were more robust at 48 vs. 24 h (Supplementary Figure 1A vs. Supplementary Figure 1B) and began subsiding by 72 h (data not shown), the 48 h time point was used to measure CNS-specific ear swelling in future experiments.

We next tested whether administration of PLP-CD8 would yield a suppressed DTH response, corresponding to suppressed EAE disease scores. Thus, recipient mice that received donor CD8 T cells from PLP178-191- or OVA323-339-immunized mice were challenged in the ear pinnae at 9 days post-immunization, followed by measurement of DTH at 48 h. Again, ears challenged with PBS showed minimal ear swelling regardless of the group, whereas mice receiving control CD8 T cells exhibited robust PLP-specific DTH responses (Figure 1B). Importantly, mice that received PLP-CD8 showed significant reduction in PLP-specific DTH, compared to their unprotected counterparts and almost down to swelling levels with PBS alone (Figure 1B). Together, these data indicate that PLP-CD8 suppress PLP-specific CD4 T cell responses *in vivo* during demyelinating disease.

### PLP-Specific CD8 T cells rapidly and robustly ameliorate ongoing demyelinating disease progression

**Figure 2:**
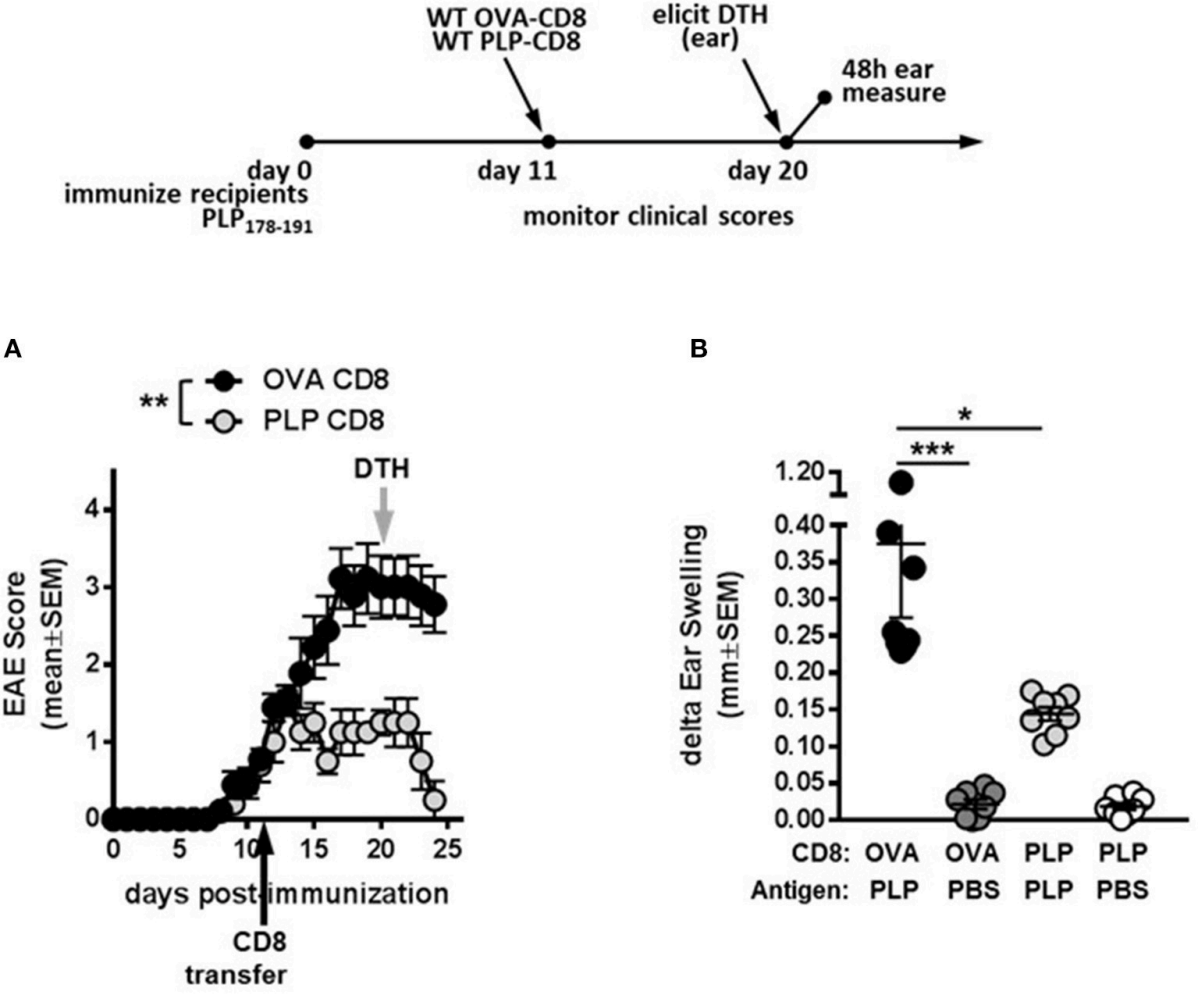
PLP-specific CD8 T cells successfully treat ongoing demyelinating disease. **(A)** Recipient mice were immunized with PLP178-191 to induce EAE and disease scores were monitored. At day 11 post-immunization (post-disease onset), OVA-CD8 or PLP-CD8 were transferred (black arrow). *n* = 8 per group. **(B)** At day 20 post-immunization (gray arrow in **A)**, ear pinnae were injected with PLP178-191 peptide or vehicle control (PBS). Ear swelling was measured at 48 h post-ear challenge. *n* = 8 per group. *p < 0.05; **p < 0.01; ***p < 0.001.

To test whether PLP-CD8 could treat ongoing disease, mice were immunized with PLP178-191/CFA to induce EAE (day 0). Donor CD8 T cells from OVA323-339- or PLP178-191-immunized mice were subsequently transferred into these mice at day 11 post-immunization (post-disease onset). Compared to the control OVA-CD8-treated group, PLP-CD8 significantly altered ongoing disease progression within 48 h, with significant reduction in EAE scores, almost eliminating clinical symptoms (Figure 2A). Likewise, when recipient ear pinnae were challenged on day 20 post-immunization (9 days post-CD8 treatment), control mice developed a robust DTH reaction to PLP178-191, whereas mice treated with PLP-CD8 exhibited a significantly reduced DTH response (Figure 2B), matching their diminished disease scores. These data suggest that PLP-CD8 can rapidly exert a suppressive program on pathogenic biology *in vivo*.

### PLP-Specific CD8 T cells rapidly suppress PLP-Specific CD4 T cell responses *in vivo*

**Figure 3:**
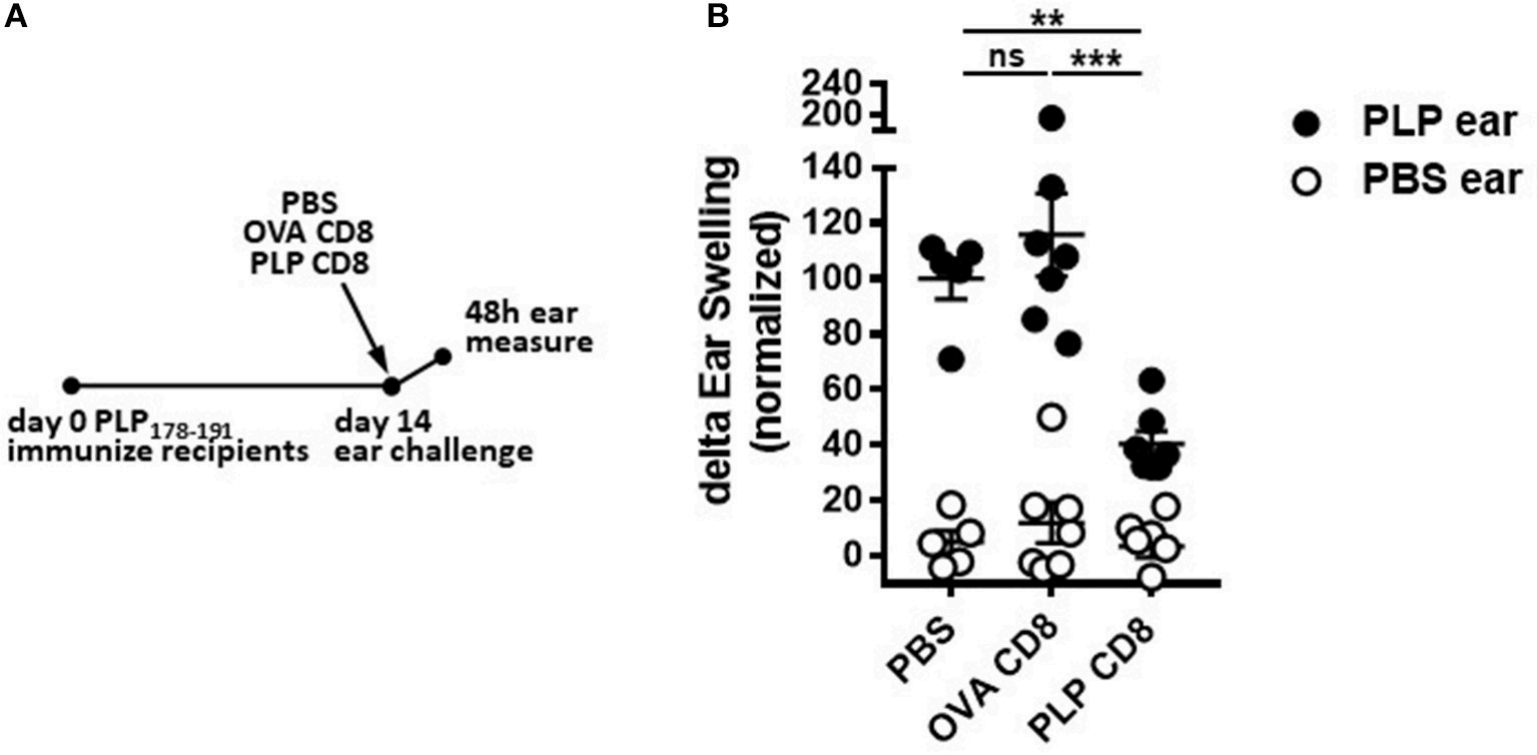
PLP-specific CD8 T cells rapidly suppress PLP-specific CD4 T cell responses *in vivo*. Mice were immunized with PLP178-191/CFA on day 0. At day 14, these mice were injected i.v. with either PBS or adoptively transferred OVA-CD8 or PLP-CD8. On the same day, the ear pinnae were challenged with either PLP178-191 peptide or PBS as a control. Ear swelling was measured at 48 h and normalized to control (untransferred) group swelling mean. *n* = 6–7 per group. Data are representative of two replicates. **p < 0.01; ***p < 0.001.

Given that PLP-CD8 altered the trajectory of ongoing demyelinating disease progression within just 2 days of treatment (Figure 2A), we hypothesized that PLP-CD8 could rapidly suppress PLP-specific CD4 T cell responses *in vivo*. To test this, mice were immunized with PLP178-191/CFA and treated i.v. with PBS, OVA-CD8, or PLP-CD8 at day14. *On the same day*, ear pinnae were challenged with vehicle control (PBS) or PLP178-191 peptide and ear measurements were performed at 48 h to assess *in vivo* DTH responses. Mice receiving no CD8 transfer (PBS control) or control OVA-CD8 developed the expected robust DTH response to PLP178-191 peptide (Figure 3). Interestingly, however, immune mice receiving PLP-CD8 exhibited a significant reduction in ear swelling compared to the control groups (Figure 3). These data suggest that CNS-CD8 suppress CNS-specific CD4 T cell responses *in vivo* in a rapid timeframe, a process that begins essentially on the same day as the CD8 T cells are transferred into the animals.

### Perforin production by PLP-Specific CD8 T cells is not required for rapid suppression of CD4 T cell responses *in vivo*

**Figure 4:**
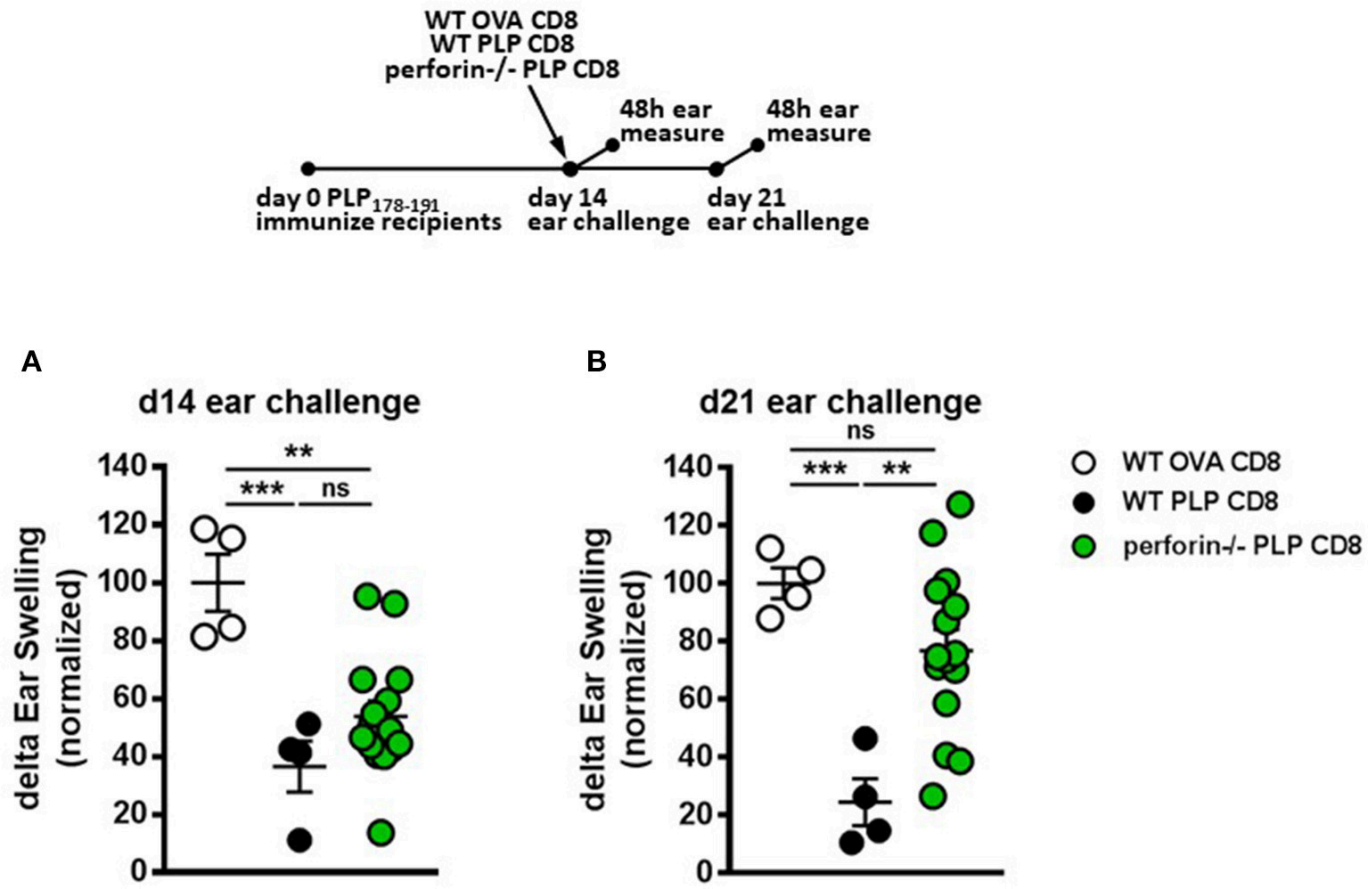
Perforin production by PLP-specific CD8 T cells is not required for rapid suppression of PLP-specific CD4 T cell responses *in vivo* but is required for late phase suppression. **(A)** Mice were immunized with PLP178-191/CFA on day 0. On day 14, mice were treated with either WT OVA-CD8, WT PLP-CD8, or perforin–/– PLP-CD8. On the same day, ears were challenged with PLP178-191 peptide. Ear swelling was measured at 48 h. **(B)** In the identical setup as **(A)**, ears were challenged at day 21 (7 days post-CD8 treatment) and swelling was measured at 48 h. OVA-CD8 *n* = 4; WT PLP-CD8 *n* = 4; perforin–/– PLP-CD8 *n* = 15. Data are representative of two replicate experiments. **p < 0.01; ***p < 0.001.

We have previously demonstrated that CNS-CD8 utilize perforin production to protect mice from severe EAE disease (11, 14). Given the rapid suppressive response by PLP-CD8 observed in the current study (Figure 3), we hypothesized that this effect might be a result of perforin-mediated cytotoxic killing of immune targets, such as CD4 T cells, a mechanism we have shown to be important in CD8-mediated suppression (11, 16). To test this, we utilized an experimental setup similar to that in Figure 3, where PLP178-191-immunized mice were treated with either WT or perforin–/– PLP-CD8 at day 14. The control group received WT OVA-CD8. On the same day, ears were challenged with PLP178-191 peptide and measured at 48 h. Control OVA-CD8 recipients developed a robust DTH response and the expected suppressed DTH response was seen in mice receiving WT PLP-CD8 (Figure 4A). Surprisingly, perforin–/– PLP-CD8 resulted in similar suppression of DTH compared to the WT group (Figure 4A), indicating that perforin was not a required effector pathway to mediate rapid CD4 suppression *in vivo*.

To test whether perforin production was required for the late DTH suppression (“maintenance phase”), ears were challenged 21 days post-immunization (i.e., 7 days post-CD8 transfer). Again, mice transferred with OVA-CD8 showed a continued robust DTH response at this stage and mice receiving WT PLP-CD8 maintained their suppressed DTH (Figure 4B). Interestingly, compared to their WT counterparts, perforin–/– PLP-CD8 failed to optimally maintain their DTH suppression (Figure 4B). When change in suppression was directly compared over time in individual mice, it was clear that while no change was observed in the OVA-CD8 or PLP-CD8 groups, DTH responses in mice receiving perforin–/– CD8 T cells were significantly less suppressed at day 21 (Supplementary Figure 2A), indicating a recovery of CD4 responses to PLP178-191. These data suggest that there is a requirement for perforin production by PLP-CD8 to maintain longer term suppression of CD4 T cell responses *in vivo*.

### IFNγ production is required for rapid suppression of CD4 T cell responses by PLP-Specific CD8 T cells *in vivo* but is not required for delayed suppression

In addition to perforin, IFNγ production by CNS-CD8 is required to mediate their ameliorative effects on EAE (11). In contrast, IL-10 or IL-4 production is not required (11). Therefore, we tested whether IFNγ production was necessary for rapid DTH suppression by PLP-CD8. Groups of recipient mice were immunized with PLP178-191 and adoptively transferred 14 days later with either control WT OVA-CD8, or WT or IFNγ-/– PLP-CD8. DTH responses were elicited and measured, both at the early (same day challenge) and late (7 days later) time points, similar to the approach in Figures 4A,B.

**Figure 5:**
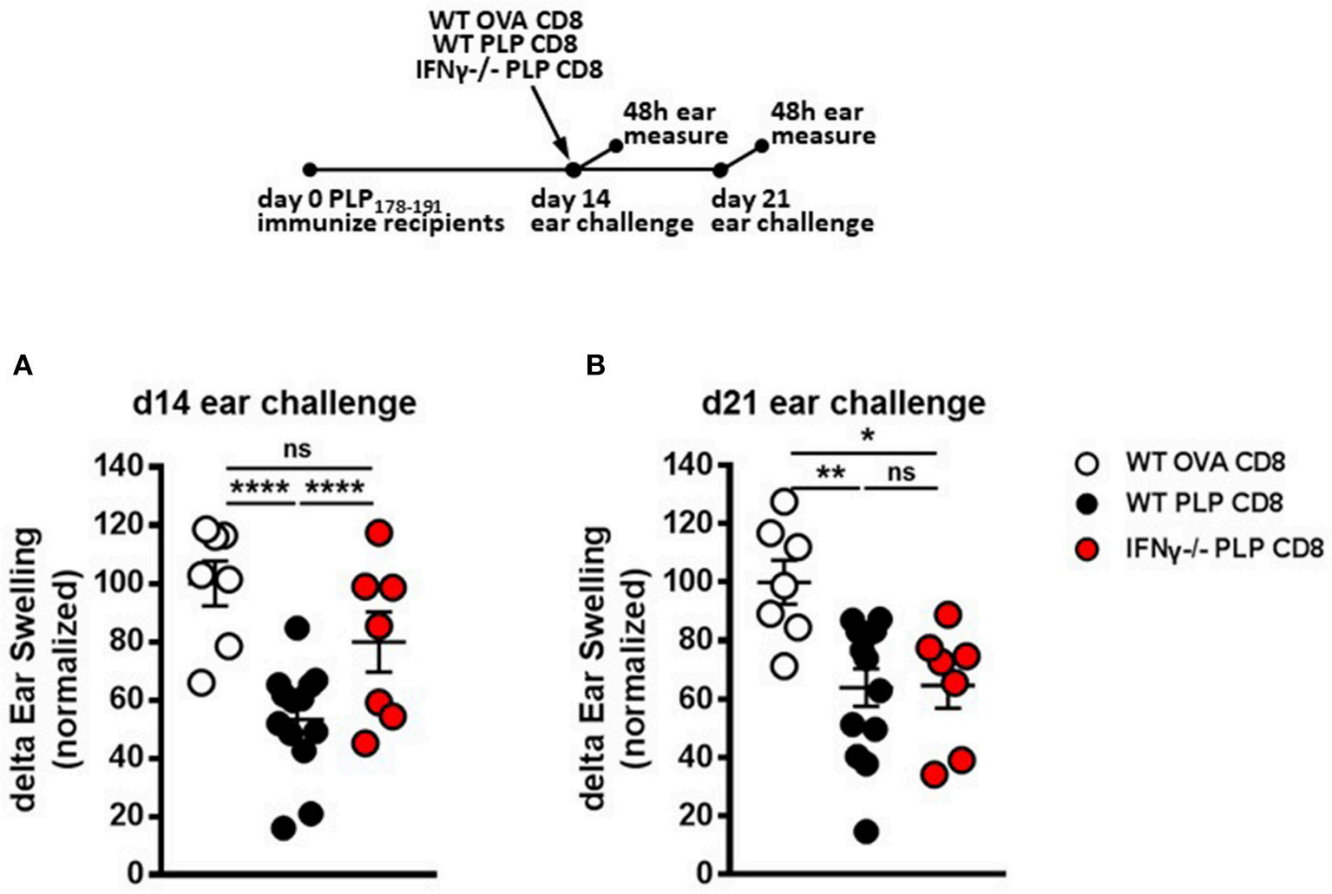
IFNγ production by PLP-specific CD8 T cells is required for rapid suppression of PLP-specific CD4 T cell responses *in vivo* but not for long-term maintenance of suppression. **(A)** Mice were immunized with PLP178-191/CFA on day 0. On day 14, mice were treated with either WT OVA-CD8, WT PLP-CD8, or IFNγ–/– PLP-CD8. On the same day, ears were challenged with PLP178-191 peptide. Ear swelling was measured at 48 h. **(B)** In the identical setup as **(A)**, ears were challenged at day 21 (7 days post-CD8 treatment) and swelling was measured at 48h. OVA-CD8 *n* = 7; WT PLP-CD8 *n* = 13; IFNγ–/– PLP-CD8 *n* = 7. Data are representative of two replicate experiments. *p < 0.05; **p < 0.01; ****p < 0.0001.

As expected, mice receiving WT PLP-CD8 showed significantly suppressed DTH responses, compared to those receiving control OVA-CD8, both at the early and late time points (Figures 5A,B). Interestingly, in contrast to perforin–/– CD8 T cells (Figure 4), IFNγ–/– CD8 T cells showed the opposite dynamics, in that they failed to rapidly suppress the DTH reaction following same-day challenge (Figure 5A), but could eventually suppress a day 21-elicited DTH reaction (Figure 5B). When change in suppression was directly compared over time, DTH in mice receiving IFNγ-/– CD8 T cells was clearly more suppressed at day 21 compared to day 14 (Supplementary Figure 2B). Together, these data indicate that PLP-CD8 mediate swift suppression of CD4 T cell responses *in vivo* using IFNγ-mediated mechanisms, whereas these mechanisms are not required to mediate late suppression.

### PLP-Specific CD8 T cells use temporally distinct effector mechanisms to mediate disease suppression

Given that IFNγ (but not perforin) production by PLP-CD8 was necessary for rapid suppression of PLP-specific CD4 T cell responses *in vivo* (Figures 4A, 5A) and, conversely, perforin (but not IFNγ) production was required for optimal longer term suppression (Figures 4B, 5B), we asked whether IFNγ–/– and perforin–/– single-knockout PLP-CD8 could temporally compensate for each other's functional deficits to exert both a rapid and maintained *in vivo* suppression effect. We therefore used an admixture of IFNγ–/– plus perforin–/– single knockout PLP-CD8 to test whether this mixture could phenocopy the suppression pattern observed in the WT scenario. Following an experimental design similar to prior figures, PLP-immunized mice received either PBS, WT PLP-CD8 or a mixture of IFNγ–/– (perforin sufficient) plus perforin–/– (IFNγ sufficient) single-knockout PLP-CD8. DTH responses to PLP178-191 were elicited and measured, both at immediate (same day) and late (7 days later) time points.

**Figure 6:**
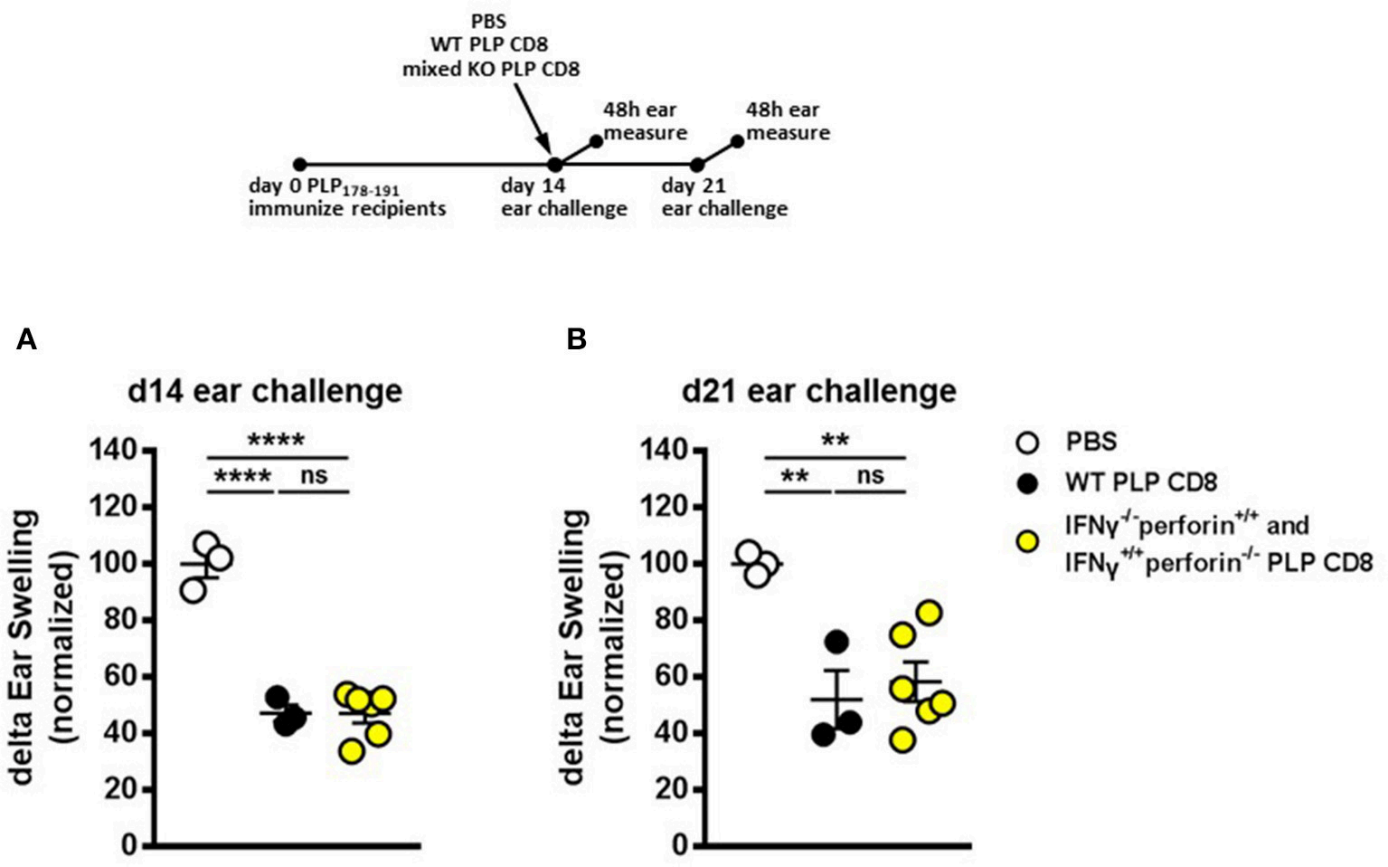
IFNγ–/– and perforin–/– PLP-specific CD8 T cells functionally compensate to rapidly suppress PLP-specific CD4 T cell responses and maintain longer term suppression *in vivo*. **(A)** Mice were immunized with PLP178-191/CFA on day 0. On day 14, mice were treated with either PBS, 5 × 10^6^ WT PLP-CD8, or an admixture of 2.5 × 10^6^ IFNγ–/– (perforin sufficient) plus 2.5 × 10^6^ perforin–/– (IFNγ sufficient) single knockout PLP-CD8. On the same day, ears were challenged with PLP178-191 peptide. Ear swelling was measured at 48 h. **(B)** In the identical setup as **(A)**, ears were challenged at day 21 (7 days post-CD8 treatment) and swelling was measured at 48h. *n* = 3–6 per group. **p < 0.01; ****p < 0.0001.

WT PLP-CD8 showed the expected rapid (Figure 6A) and maintained (Figure 6B) suppression of DTH, compared to PBS controls. Intriguingly, immune recipient mice that were adoptively transferred with both IFNγ-/– (perforin sufficient) plus perforin–/– (IFNγ sufficient) PLP-CD8 exhibited a significantly suppressed DTH response at both time points, similar to that seen with WT PLP-CD8 (Figures 6A,B). Longitudinal analysis showed that suppression was not significantly changed over time in any group (Supplementary Figure 2C). Taken together, these data suggest that IFNγ-/– and perforin–/– PLP-CD8 could compensate for each other to immediately suppress PLP-specific CD4 T cell responses and maintain suppression *in vivo*.

**Figure 7:**
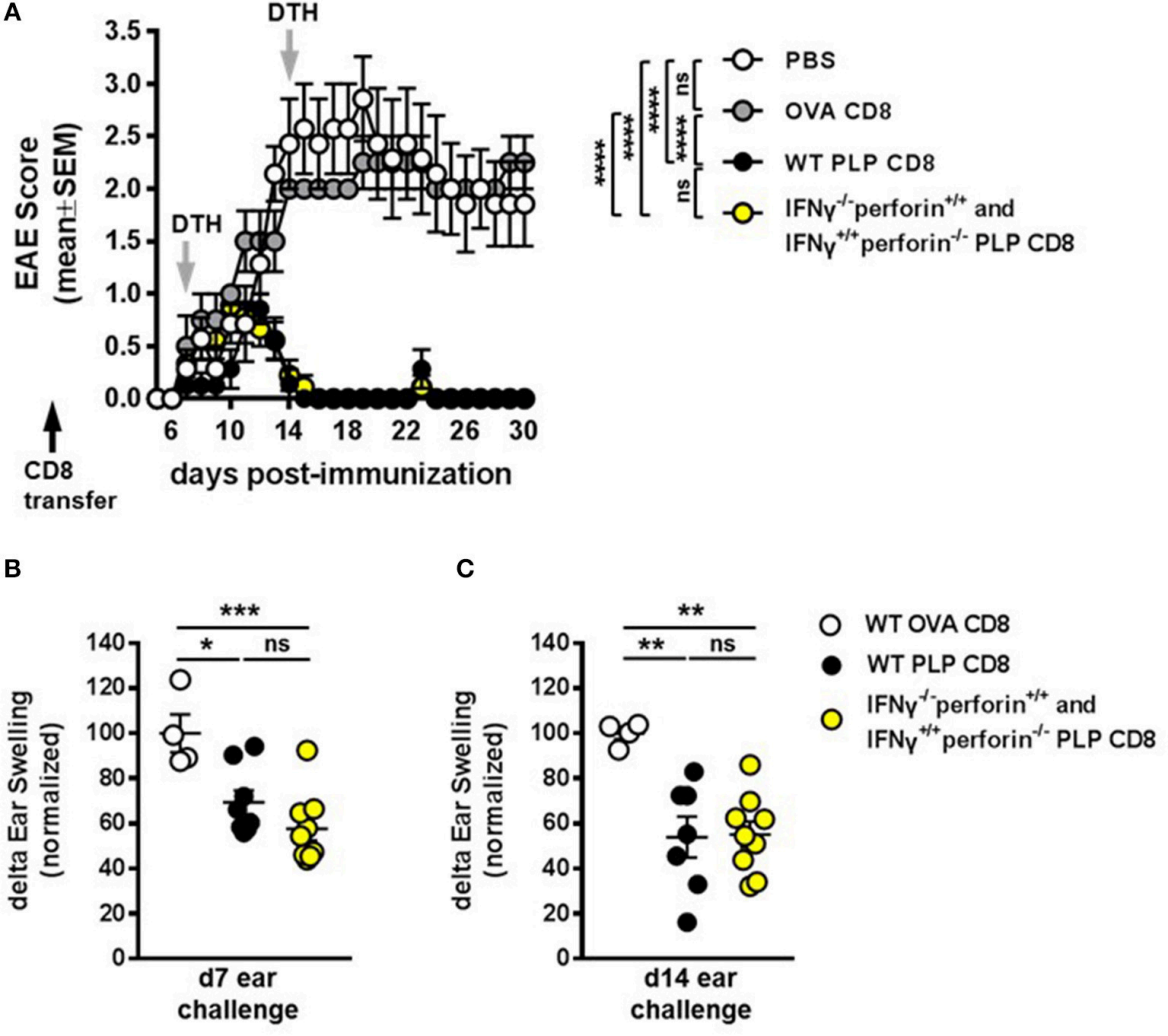
IFNγ–/– and perforin–/– PLP-specific CD8 T cells functionally compensate to protect mice from EAE. **(A)** On day −1 (black arrow), mice received either PBS, 5 × 10^6^ WT PLP-CD8, or 2.5 × 10^6^ IFNγ–/– (perforin sufficient) plus 2.5 × 10^6^ perforin–/– (IFNγ sufficient) single knockout PLP-CD8. The following day, mice were immunized with PLP178-191/CFA to induce EAE and disease scores were monitored. **(B)** On day 7 post-immunization (first gray arrow), right ears were challenged with PLP178-191 peptide and measured at 48 h. **(C)** On day 14 (second gray arrow), left ears were injected with PLP178-191 peptide and measured at 48 h. PBS control *n* = 7, OVA-CD8 *n* = 4; WT PLP-CD8 *n* = 7; mixed knockout PLP-CD8 *n* = 9. *p < 0.05; **p < 0.01; ***p < 0.001; ****p < 0.0001.

As mentioned above, our previous work has demonstrated that neither IFNγ-/– nor perforin–/– CNS-CD8 are capable of protecting mice from EAE, using MOG-specific CD8 T cells (11). To formally confirm that this was also the case for PLP-specific CD8 T cells, we performed experiments using IFNγ-/– or perforin–/– PLP-CD8. As expected, PLP-CD8 deficient in either of these molecules were not capable of suppressing EAE, unlike WT PLP-CD8 (Supplementary Figure 3). Given that PLP-CD8 lacking perforin can functionally compensate for cells lacking IFNγ, and vice versa, in order to effect and maintain suppression of PLP-specific CD4 T cell responses *in vivo* (Figure 6), we hypothesized that a mixture of adoptively transferred IFNγ-/– plus perforin–/– single knockout PLP-CD8 could successfully protect mice against EAE disease. To test this, WT donor CD8 T cells from PLP178-191- or OVA323-339-immunized mice or a mixture of IFNγ-/– plus perforin–/– single knockout PLP-CD8 were adoptively transferred into groups of naïve C57BL/6J mice. An additional control group received PBS alone. The following day, recipient mice were immunized with PLP178-191 to induce EAE and disease progression was monitored. Compared to the PBS and OVA-CD8 control groups, WT PLP-CD8 significantly protected mice from EAE (Figure 7A). Notably, mice receiving IFNγ/perforin mixed single knockout PLP-CD8 were equally effective in robustly protecting mice from EAE disease (Figure 7A), suggesting a successful mechanistic compensation for the lack of protective function from either CD8 type alone.

To further confirm that the compensation effect on PLP-specific CD4 T cell responses occurs during disease progression, DTH responses were elicited at day 7 post-immunization and measured at 48 h. Again, DTH responses were significantly suppressed in recipients of WT PLP-CD8 compared to OVA-CD8 controls (Figure 7B). Importantly, mice that received adoptively transferred IFNγ-/- plus perforin-/- single knockout PLP-CD8 also exhibited a similarly reduced DTH reaction (Figure 7B). To test whether this effect was maintained over time during disease progression, ear challenges were performed on day 14 and measured at 48 h. Consistent with maintained EAE suppression observed in Figure 7A, and maintained DTH suppression in Figure 6B, mice receiving the mixture of single-knockout PLP-CD8 continued to show significantly suppressed *in vivo* DTH responses, similar to those receiving WT PLP-CD8 (Figure 7C). Taken together, these results demonstrate that PLP-CD8 employ an ordered regulatory program over a number of days *in vivo* to suppress pathogenic PLP-specific CD4 T cell responses and inhibit demyelinating disease.

## Discussion

Despite the lack of etiological explanation, it has been long appreciated that autoreactive immune cells in their various proinflammatory states are at the core of driving chronic demyelinating CNS diseases like MS. Consequently, dissecting how immunoregulatory responses combat this immunopathology *in vivo* is essential to understanding biological mechanisms and potential immunotherapies. The murine model of demyelinating disease, EAE, has been instrumental in this regard, as it serves as a testable arena for immunomodulation.

We have now demonstrated that in the wild-type setting, unlike their CD4 counterparts, CNS-CD8 not only fail to transfer or exacerbate demyelinating disease, but are unexpectedly protective against EAE (7). We have shown that this is true of CD8 responses induced by both peptide immunization (involving cross-presentation of an extrinsic antigen) and by infection with CNS sequence-encoding intracellular bacteria (Listeria) (7, 11–14). These findings are further underscored by our observations that MS patients undergoing an acute relapse exhibit an immunoregulatory defect in their CD8 T cell population (15, 16). Thus, interrogating the functional mechanisms and autoregulatory potential of the most potently suppressive CNS-CD8 subsets *in vivo* is of high interest and potential therapeutic relevance.

Recent work from our lab has demonstrated that PLP-CD8 are extremely potent suppressors of EAE in both the B6 and SJL models (13, 14) (and confirmed here in Figure 1A). Given the identical homology between murine and human PLP (17), we focused on these autoregulatory cells in the current study. During protection against EAE (Figure 1A), PLP-CD8 significantly suppressed PLP-specific CD4 T cell responses as read by DTH (Figure 1B). These cells also have therapeutic potential, as they strikingly ameliorated ongoing EAE, essentially eliminating clinical symptoms (Figure 2A). The rapidity (around 48 h; Figure 2A) with which PLP-CD8 reversed the EAE disease course led us to consider whether these cells were immediately suppressing PLP-specific CD4 T cell responses, an effect inherent to disease amelioration (Figure 2B). As shown in Figure 3, this was in fact the case, where PLP-CD8 quickly and significantly reduced DTH reactions elicited on the same day as the CD8 T cell transfer. This is suggestive of immediate suppressive action *in vivo*, and may indicate rapid targeting and elimination/modulation of pathogenic targets.

In previously published studies (11, 14), we have shown that CNS-CD8 are classically MHC Class Ia-restricted and require IFNγ as well as perforin, but not IL-4 or IL-10 production to mediate their disease suppressive effects. Furthermore, there is evidence of cytotoxic elimination of immune targets by these cells (7). Therefore, we first thought that the fast suppression of DTH responses might be an effect of rapid elimination of CD4 T cells by a cytotoxic mechanism. We thus tested the requirement for perforin in this setting. Surprisingly, PLP-CD8 deficient in perforin production were perfectly capable of rapidly suppressing the DTH response *in vivo* comparable to WT CD8 T cells (Figure 4A), suggesting that perforin-dependent mechanisms of regulation are ancillary for the rapid suppressive effect on CNS peptide-driven DTH. However, perforin was required for optimal longer term suppression, read at 7 days post-transfer (Figure 4B).

In contrast, the pleiotropic immunomodulatory cytokine, IFNγ, was necessary for swift suppression of PLP-specific CD4 T cell responses (Figure 5A), but was not required for longer term suppression (Figure 5B). These opposing findings, whereby IFNγ was required early and perforin was required later to effect both optimal rapid and maintained DTH suppression, is indicative of an ordered regulatory program exerted by PLP-CD8 over a number of days *in vivo*.

Since neither IFNγ-/- nor perforin-/- single knockout CNS-CD8 are capable of suppressing disease [Supplementary Figure 3 and (11)], it would be reasonably expected that double knockout PLP-CD8 would also not suppress EAE. Therefore, we did not utilize double knockout PLP-CD8 in these experiments. Instead, upon observing the distinct temporal dynamics of the single knockout cells in suppressing DTH responses, we asked whether an admixture of the single knockout cells would have a compensatory effect. Indeed, if immune mice were transferred both types of single knockout PLP-CD8 together (IFNγ-/- plus perforin-/-), the treatment phenocopied the WT scenario, where PLP-driven DTH was not only swiftly suppressed (Figure 6A), but remained suppressed over time (Figure 6B). Importantly, mice that were transferred the single knockout mixture prior to EAE induction were equally protected from EAE disease as their WT CD8 T cell-transferred counterparts (Figure 7A). Concordantly, PLP-driven DTH was equally suppressed in both instances compared to OVA-CD8 controls (Figures 7B,C).

Taken together, this study provides important insights into the *in vivo* processes that occur upon immunotherapeutic adoptive CD8 T autoregulatory cell transfer. The differential requirement for perforin and IFNγ production by PLP-CD8 with respect to timing may suggest interactions with two distinct cellular targets in the course of their immunoregulatory exertion. We have previously shown that treatment of mice with CNS-CD8 results in both the downregulation of CNS-specific CD4 T cell responses (11) as well as modulation of antigen-presenting cells (APC), particularly dendritic cells (12). These data in conjunction with the temporal findings in the current study may indicate a dualistic suppression program whereby CNS-CD8 initially utilize IFNγ to immunomodulate APC populations while cytotoxic properties are eventually required for pathogenic CD4 T cell elimination over time. Future receptor knockout and cytotoxicity studies will be vital in teasing apart dualistic regulation *in vivo*. Related to this issue are our prior observations that CNS-CD8 of different specificities require cognate antigenic stimulation *in vivo* in the context of classical MHC Class Ia molecules (11). Thus, MOG-specific CD8 are unable to suppress PLP-induced disease and, likewise, PLP-CD8 do not suppress MOG-induced EAE (13). In the context of our DTH studies, where injected antigen is presumably presented by skin APC in the ear, the results suggest a model in which antigen-specific IFNγ-mediated APC modulation may be an early event in this cascade of interactions, dependent on the presentation of the cognate antigen. Eventually, perforin-mediated cytotoxic elimination of either APC subsets or pathogenic CD4 T cells might become an essential mechanism of sustained suppression, reflected in the late DTH data. Again, based on previous observations (7, 11), these interactions also seem to require cognate antigenic presentation and may depend on acquisition of antigen by CD4 T cells through processes such as trogocytosis. Importantly, our DTH system will now allow the dissection of potential bystander suppression when antigens can be presented *in vivo* in a non-encephalitogenic manner to CNS-CD8 and CNS-CD4 of differing antigenic specificities.

IFNγ and perforin have been described to regulate antigen-specific CD8 T cell homeostasis (23). Indeed, various aspects of CD8 T cell biology (differentiation, motility, cytotoxicity, etc.) are, in part, regulated by IFNγ (24–31). Further evidence suggests that IFNγ promotes perforin-mediated killing ability in CD8 T cells (32) and that perforin-mediated control of infection is dependent on IFNγ (33). Thus, in the context of the current study, it is possible that lack of IFNγ production by CD8 T cells inhibits their immediate ability to employ immune suppressive effects *in vivo*. Importantly, these cells were not developmentally affected in the donor mice, since addition of IFNγ-replete (but perforin-deficient) PLP-CD8 resulted in robust compensation of the suppressor phenotype. Since both of the admixed cells shared the same antigenic target, it appears that IFNγ production in the vicinity of the overall CD8 T cell response is important in this process, arguing for an autocrine/paracrine mechanism. Conversely, we have also demonstrated that IFNγ receptor-deficient CNS-CD8 were capable of suppressing EAE. However, there was a clear delay before the disease suppressive effect could be observed (11), again demonstrating the early need for IFNγ-mediated potentiation, which was not needed at later stages of the disease.

Ultimately, the compensation of one knockout cell type by the other could suggest that the two effector pathways do not necessarily have to emerge from the same cell. One of the effects of IFNγ is to upregulate MHC Class I expression (34, 35). Thus, one possible interplay is that IFNγ may be required for Class I upregulation, which in turn makes the target cells more susceptible to perforin-mediated elimination. Alternatively, it may be that early exposure of CD8 T cells to IFNγ will elicit a quick burst of perforin, whereas later in time when CD8 T cells are less responsive to IFNγ signaling (29, 30), their perforin production is enhanced by any number of other cellular interplays, namely MHC contact and TCR stimulation via the acquisition of a target cell. This link between IFNγ and perforin in CD8 T cell-mediated regulation of EAE requires further study.

To summarize, we offer here important insights into the *in vivo* regulatory mechanics of CNS-CD8 by demonstrating that these cells utilize a temporally distinct regulatory program involving IFNγ and perforin production to suppress pathogenic PLP-specific CD4 T cell responses during protection against EAE disease. Going forward, elucidating the complex cellular interplay that occurs during CNS-CD8 adoptive transfer, as well as the autoregulatory functions and temporal mechanics involved, will be critical for interrogating these cells' effectiveness as a potential immunotherapeutic for MS patients.

## Data availability statement

All relevant datasets generated for this study are included in the manuscript and the supplementary files.

## Ethics statement

This study was carried out in accordance with the PHS Policy on Humane Care and Use of Laboratory Animals, the Guide for the Care and Use of Laboratory Animals, and the NIH Office of Laboratory Animal Welfare. The protocol was approved by the University of Iowa's Office of Institutional Animal Care and Use Committee.

## Author contributions

AWB and NJK contributed to the conception and design of the study. AWB and AAB performed the experiments. AWB organized the datasets and wrote the first draft of the manuscript. All authors contributed to manuscript revision, read, and approved the submitted version.

### Conflict of interest statement

The authors declare that the research was conducted in the absence of any commercial or financial relationships that could be construed as a potential conflict of interest.
