# Supplementary material for: Early IFNγ-Mediated and Late Perforin-Mediated Suppression of Pathogenic CD4 T Cell Responses Are Both Required for Inhibition of Demyelinating Disease by CNS-Specific Autoregulatory CD8 T Cells
Source: Front Immunol. 2018 Oct 9;9:2336. doi: 10.3389/fimmu.2018.02336 (PMC6189364; doi:10.3389/fimmu.2018.02336)
Supplement: Supplementary file 1 [file Data_Sheet_1.PDF]

Supplementary Material

Early IFNγ-mediated and late perforin-mediated suppression of pathogenic CD4 T cell responses are both required for inhibition of demyelinating disease by CNS-specific autoregulatory CD8 T cells

Alexander W. Boyden, Ashley A. Brate, *Nitin J. Karandikar

***Correspondence:** Nitin J. Karandikar: nitin-karandikar@uiowa.edu

Supplementary Figures

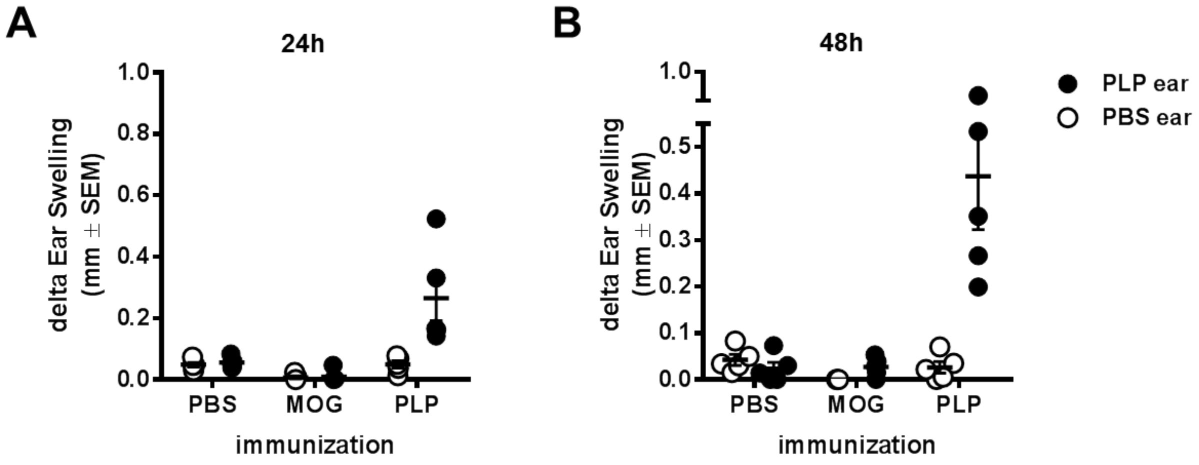

Supplementary Figure 1. PLP178-191/CFA immunization elicits a PLP178-191 peptide-specific DTH response. Mice were immunized with PBS/CFA, MOG35-55/CFA, or PLP178-191/CFA on day 0. At a later time point, ear pinnae were injected with PLP178-191 peptide or PBS as a control. Ear swelling was measured at **(A)** 24h or **(B)** 48h. n = 5 per group.

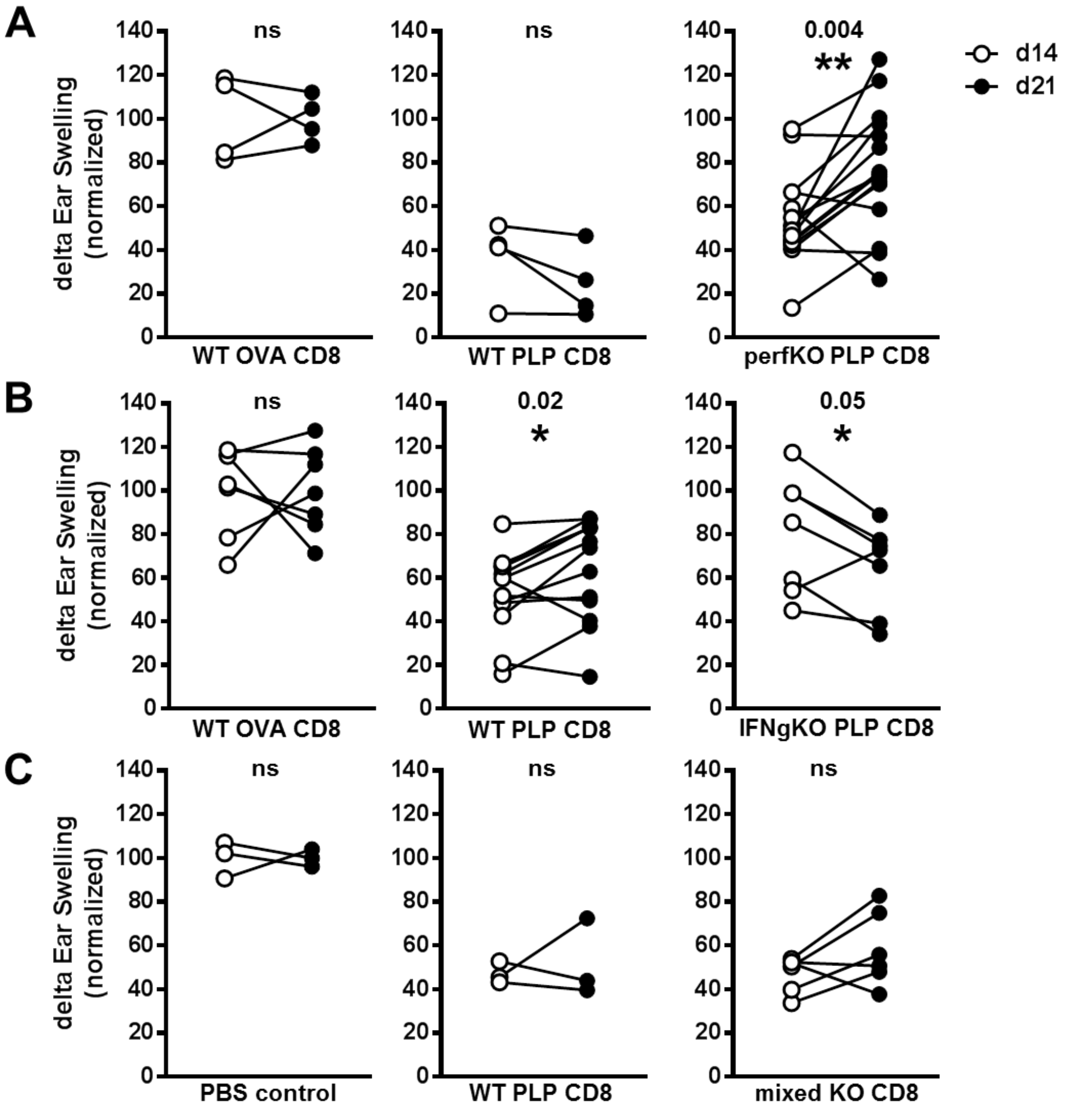

Supplementary Figure 2. Longitudinal DTH suppression analysis. Normalized suppression data from CNS-CD8-transferred mice were compared over time (day 14 vs. day 21). Individual mice are depicted as dots representing the two longitudinal time points, connected by a line. **(A)** Suppression data from Fig. 4. **(B)** Suppression data from Fig. 5. **(C)** Suppression data from Fig. 6. Data were statistically analyzed with a paired student's t-test.

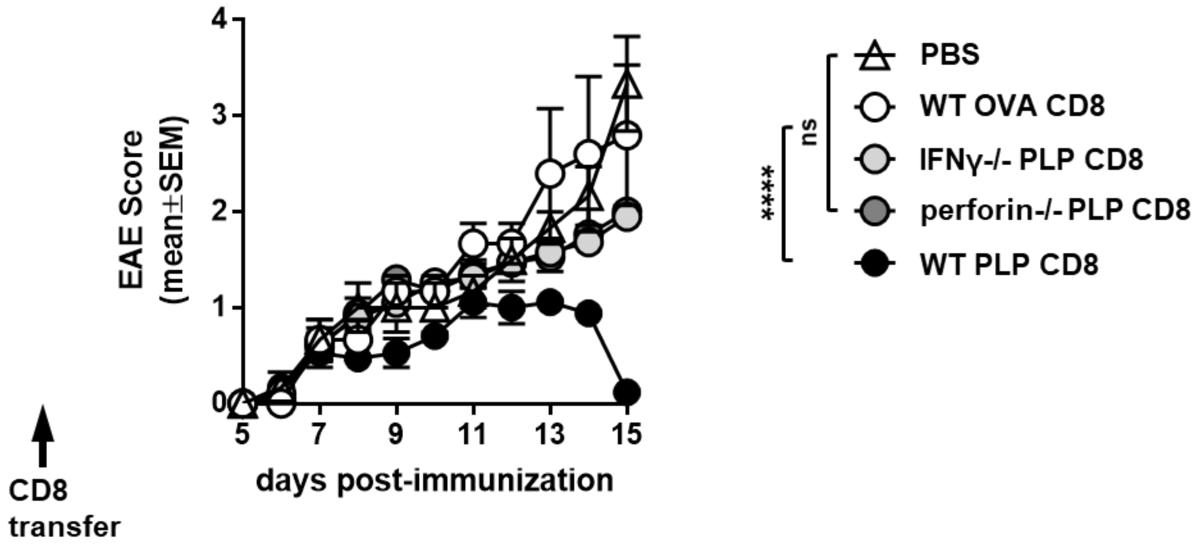

Supplementary Figure 3. IFNγ-/- and perforin-/- PLP-specific CD8 T cells fail to protect mice from EAE disease. On day -1 (black arrow), WT OVA-CD8 (n=6), IFNγ-/- PLP-CD8 (n=19), perforin-/- PLP-CD8 (n=17), or WT PLP-CD8 (n=16) were adoptively transferred to naïve recipients. An additional control group received PBS alone (n=6). The following day, mice were immunized with PLP178-191/CFA to induce EAE and disease scores were monitored. **** $p<0.0001$.
